# Virus-virus interactions alter the mechanical transmissibility and host range of begomoviruses

**DOI:** 10.3389/fpls.2023.1092998

**Published:** 2023-06-02

**Authors:** Ho-Hsiung Chang, Deri Gustian, Chung-Jan Chang, Fuh-Jyh Jan

**Affiliations:** ^1^ Department of Plant Pathology, National Chung Hsing University, Taichung, Taiwan; ^2^ Department of Plant Pathology, University of Georgia, Griffin, GA, United States; ^3^ Advanced Plant and Food Crop Biotechnology Center, National Chung Hsing University, Taichung, Taiwan

**Keywords:** begomovirus, virus-virus interaction, movement protein, mechanical transmissibility, host range

## Abstract

**Introduction:**

Begomoviruses are mainly transmitted by whiteflies. However, a few begomoviruses can be transmitted mechanically. Mechanical transmissibility affects begomoviral distribution in the field.

**Materials and methods:**

In this study, two mechanically transmissible begomoviruses, tomato leaf curl New Delhi virus-oriental melon isolate (ToLCNDV-OM) and tomato yellow leaf curl Thailand virus (TYLCTHV), and two nonmechanically transmissible begomoviruses, ToLCNDV-cucumber isolate (ToLCNDV-CB) and tomato leaf curl Taiwan virus (ToLCTV), were used to study the effects of virus-virus interactions on mechanical transmissibility.

**Results:**

*Nicotiana benthamiana* and host plants were coinoculated through mechanical transmission with inoculants derived from plants that were mix-infected or inoculants derived from individually infected plants, and the inoculants were mixed immediately before inoculation. Our results showed that ToLCNDV-CB was mechanically transmitted with ToLCNDV-OM to *N. benthamiana*, cucumber, and oriental melon, whereas ToLCTV was mechanically transmitted with TYLCTHV to *N. benthamiana* and tomato. For crossing host range inoculation, ToLCNDV-CB was mechanically transmitted with TYLCTHV to *N. benthamiana* and its nonhost tomato, while ToLCTV with ToLCNDV-OM was transmitted to *N. benthamiana* and its nonhost oriental melon. For sequential inoculation, ToLCNDV-CB and ToLCTV were mechanically transmitted to *N. benthamiana* plants that were either preinfected with ToLCNDV-OM or TYLCTHV. The results of fluorescence resonance energy transfer analyses showed that the nuclear shuttle protein of ToLCNDV-CB (CBNSP) and the coat protein of ToLCTV (TWCP) localized alone to the nucleus. When coexpressed with movement proteins of ToLCNDV-OM or TYLCTHV, CBNSP and TWCP relocalized to both the nucleus and the cellular periphery and interacted with movement proteins.

**Discussion:**

Our findings indicated that virus-virus interactions in mixed infection circumstances could complement the mechanical transmissibility of nonmechanically transmissible begomoviruses and alter their host range. These findings provide new insight into complex virus-virus interactions and will help us to understand the begomoviral distribution and to reevaluate disease management strategies in the field.

## Introduction

To study virus-host interactions, viral infections are usually conducted with the use of a single infection in the laboratory. Viral infections in nature can occur as part of mixed infections involving more than one type of virus. The results of a field survey between 2008 and 2009 in Taiwan indicated that more than 41% of virus-infected tomato were coinfected by more than two different viruses (Tsai et al., 2011). A number of important plant viral diseases are the result of the interactions of causal agents (Xu et al., 2022b). For instance, a large outbreak of maize lethal necrosis in sub-Saharan East Africa, Southeast Asia, and Southern America (Redinbaugh and Stewart, 2018) was the outcome of the coinfection between maize chlorotic mottle virus (MCMV) and one of several viruses from the *Potyviridae* family. The coinfection of viruses results in frequent maize death and negligible yields. Although important advances have been made toward understanding the biology of individual viruses, little attention has been given to the investigation of intrahost interactions during viral coinfections.

Multiple infections cause a variety of intrahost virus-virus interactions. These interactions can result in the development of novel phenomena and alter the genetic structure of viruses (Syller, 2012). A study of virus-virus interactions may be important for the understanding of viral pathogenesis and evolution and for the development of control strategies. DaPalma et al. (2010) classified potential virus-virus interactions into three categories: (1) direct interactions that occur in nucleic acids or proteins of one virus physically interacting with those of another virus, (2) environmental interactions that result from the alterations of the host environment created by coinfection, and (3) immunological interactions that are unique in organisms equipped with an adaptive immune system. Since plants do not have an immune system like that of animals, virus-virus interactions of the first two categories mentioned above can therefore be investigated in plants.

Takács et al. (2014) identified five *in planta* virus-virus interactions based on various plant responses, including cross-protection, synergistic and antagonistic interactions, recombination, heteroencapsidation, and gene silencing (Takács et al., 2014). Cross-protection, first described by McKinney (Mckinney, 1929), has been successfully applied for controlling critical viral diseases in the field (Oberemok et al., 2021; Tran et al., 2022; Xu et al., 2022a). The mechanism of cross-protection involves several phases. One mechanism of cross-protection is protein-mediated resistance through interference with the uncoating of the second challenging virus (Lu et al., 1998; Lin et al., 2007). Another mechanism involved in cross-protection is posttranscriptional gene silencing in a sequence homology-dependent manner against the second related viral strain (Ziebell and Carr, 2010; Pechinger et al., 2019). Both mechanisms illustrate direct virus-virus interactions, viral protein to viral genome interactions and viral RNA to viral RNA interactions. Environmentally indirect virus-virus interactions through the sequestration of essential host factors responsible for the propagation and movement of the challenging virus have been proposed as a possible mechanism for cross-protection (Folimonova, 2012).

Synergism is a phenomenon in which a mixed infection of two or more viruses leads to an increased accumulation of at least one virus that induces symptoms with increased severity. Mechanisms of this synergism have been proposed: viral proteins of one invading virus facilitate the movement of another coinfecting virus (Karyeija et al., 2000) or suppress plant defense against viruses (Fukuzawa et al., 2010; Redinbaugh and Stewart, 2018) through indirect virus-virus interactions. Synergistic interactions have been reported in unrelated viruses, such as those of different begomovirus species (Chakraborty et al., 2008; Alves-Júnior et al., 2009; Rentería-Canett et al., 2011; Sufrin-Ringwald and Lapidot, 2011) and of two viruses belonging to different genera of the family *Potyviridae* (Tatineni et al., 2010). Syller classified the phenomenon of antagonistic interactions into two types: superinfection exclusion (also known as cross-protection) and mutual exclusion (Syller, 2012). In the situation of mutual exclusion, two different subgroups of CMV colonized different cells in coinfected cowpea plants. The spatial separation of mutual exclusion reduces the opportunities for competition between each variant and the recombination that may generate genetic variation (Elena et al., 2011). There are two other types of direct virus-virus interactions that may change the characteristics of viruses in the field. Genetic recombination, observed in both plant RNA and DNA viruses, allows coinfecting viruses to propagate in a parasexual reproductive manner and promotes the evolution of viruses (Sztuba-Solińska et al., 2011; Lefeuvre and Moriones, 2015). Heteroencapsidation is the encapsidation of the genome of one virus by the coat protein (CP) of another virus. The viral CP is usually involved in long-distance viral movement around infected plants and in insect vector transmission. Heteroencapsidation often occurs under mixed-infection conditions to enhance systemic movement and provide insect transmissibility to a coinfecting virus (Bourdin and Lecoq, 1991; Takács et al., 2014).

The genus *Begomovirus*, belonging to the family *Geminiviridae*, is the largest genus in the entire virosphere and contains more than 440 species (Fiallo-Olivé et al., 2021). The genome of begomoviruses is composed of one (monopartite) or two (bipartite) circular single-stranded DNA genomes: DNA-A and DNA-B. Plants can be infected with multiple begomoviruses (Nawaz-Ul-Rehman and Fauquet, 2009; Kanakala et al., 2013). A high level of mixed infection increases opportunities for *in vivo* virus-virus interactions. The commonly observed virus-virus interactions of begomoviruses are genome reassortment and recombination; as such, begomoviruses represent the largest virus group (Hanley-Bowdoin et al., 2013; García-Arenal and Zerbini, 2019). Begomoviruses are mainly transmitted in the field. Heteroencapsidation between begomoviruses was shown to influence the efficiency of whitefly transmission (Kanakala et al., 2013). However, reports about virus-virus interactions of begomoviruses, especially at the viral protein level, are limited. Therefore, interactions associated with viral protein levels warrant further investigation.

Understanding the mechanical transmissibility of a virus is crucial for the study of the spread and control of that virus. In our previous study, we reported that the determinant of mechanical transmissibility is the begomoviral movement protein (MP) (Lee et al., 2020). In this study, we tried to understand whether virus-virus interactions affect begomoviral mechanical transmissibility. Four begomoviruses, namely, two nonmechanically transmissible begomoviruses, tomato leaf curl New Delhi virus-cucumber isolate (ToLCNDV-CB) and tomato leaf curl Taiwan virus (ToLCTV), and two mechanically transmissible begomoviruses, ToLCNDV-oriental melon isolate (ToLCNDV-OM) and tomato yellow leaf curl Thailand virus (TYLCTHV), were selected for analyses. Our results showed that virus-virus interactions during viral coinfection complemented mechanical transmissibility and altered the host range of nonmechanically transmissible begomoviruses.

## Materials and methods

### Plant materials

The plants used in this study included *N. benthamiana*, cucumber (*Cucumis sativus* cv. MY02363), oriental melon (*Cucumis melo* var. *makuwa* cv. Silver Light), and tomato (*Solanum lycopersicum* cv. ANT22). Cucumber and oriental melon seeds were purchased from Known-You Seed Company (Kaohsiung, Taiwan). Tomato seeds were kindly provided by Dr. Wen-Shi Tsai (National Chiayi University, Chiayi, Taiwan). The resulting seedlings were transplanted into pots one week after germination. Plants that had been transplanted for one to five weeks were subjected to virus inoculation (one week for cucumber, oriental melon and tomato and five weeks for tobacco plants). The inoculated plants were maintained in a greenhouse located at the National Chung Hsing University (Taichung, Taiwan).

### Agroinfiltration and virus inoculum preparation

The preparations of the infectious clones of ToLCNDV-CB, ToLCNDV-OM, ToLCTV, and TYLCTHV were carried out as described in our previous studies (Chang et al., 2010; Tsai et al., 2011; Lee et al., 2020). Agroinfiltration was conducted with a previously described method (Chang et al., 2022), with some modifications. A single colony was picked and cultured overnight at 28°C in 4 ml of lysogeny broth (LB) media supplemented with appropriate antibiotics (50 mg/ml kanamycin for infectious clones of ToLCNDV-CB DNA-A and DNA-B, TYLCTHV DNA-A and DNA-B, ToLCTV and ToLCNDV-OM DNA-B and 50 mg/ml gentamycin for ToLCNDV-OM DNA-B). One milliliter of the overnight culture was transferred into 10 ml of LB media supplemented with appropriate antibiotics plus 100 μM acetosyringone (AS) and incubated further at 28°C until the bacterial density reached an OD600 of 1.0 (Multiskan FC, Thermo Fisher Scientific, Inc., Massachusetts). The culture was centrifuged at 5,000 × *g* (Rotor JA25.5, Beckman Coulter, Inc., California) for 10 min, after which the *Agrobacterium* cells were resuspended in 20 ml of infiltration media (10 mM 2-N morpholino-ethanesulfonic acid, 10 mM MgCl2, and 100 μM AS) and then incubated at room temperature for 2 h before infiltration. Agrobacteria carrying the infectious DNA-A or DNA-B constructs were coinjected in equal amounts into leaves of *N. benthamiana*.

Viral inocula were prepared in single and mixed-infection types. Infectious clones for one virus were agroinfiltrated into *N. benthamiana* for the single virus-infecting inoculum. Infectious clones for two viruses (one of which was a mechanically transmissible virus, such as ToLCNDV-OM and TYLCTHV, and the other was a nonmechanically transmissible virus, such as ToLCNDV-CB and ToLCTV) were mixed in equal amounts for agroinfiltration as mixed-infection inocula, namely, “virus A/virus B”.

### Mechanical inoculation

Symptomatic leaves of agroinfiltrated *N. benthamiana* plants were collected as inocula for mechanical inoculation. The leaf samples were ground in 10 mM potassium phosphate buffer (pH 7.0) at a 1:20 ratio (weight/volume). The resultant sap was inoculated onto *N. benthamiana*, cucumber, oriental melon, and tomato plants by rubbing with carborundum powder (400-500 mesh). Afterward, the inoculated leaves were washed with distilled water to remove the inoculation buffer and carborundum powder from the leaf surface. The inoculated plants were subsequently grown in an insect-free greenhouse. Two to four weeks after inoculation, systemic leaf samples of the mechanically inoculated plants (two weeks for tobacco, cucumber and oriental melon and four weeks for tomato) were collected for virus detection.

Mechanical inoculation involved three inoculation types. The first one was a single-virus inoculation type with inocula derived from the diseased plants infected by only one virus isolate; the plants were labeled “virus A” or “virus B”. The second one was a mixed-infection inoculation type with inocula derived from the diseased plants infected by two different viruses; the plants were labeled “virus A/virus B”. The third type involved two viruses coinoculated (coinoculation type) with an inoculum mixture derived from two individual diseased plants infected by different viruses; the plants inoculated in this manner were labeled “virus A+virus B”.

### DNA extraction

Plant total DNA was extracted following the methods of Lin et al. (2012) *via* microprep buffer containing a mixture of three kinds of buffer, namely, DNA extraction buffer [0.35 M sorbitol, 1.1 M Tris and 5 mM EDTA (pH adjusted to 7.5)], nuclei lysis buffer (1.2 M Tris, 0.05 M EDTA, 2 M NaCl and 2% CTAB), and 5% sarkosyl, at a 5:5:2 volumetric ratio. The collected leaf samples (50-100 mg per sample) were ground in 750 μl of microprep buffer. The ground sample solution was incubated at 65 °C for 30-120 min. Five hundred microliters of chloroform/isoamyl (24:1) mixture was then added to the sample solution, which was then vortexed for 0.5-1 min. The homogenized samples were then centrifuged at 12000 × *g* (Rotor F45-24-11, Centrifuge 5415D, Eppendorf, Hamburg, Germany) for 5 min. The top layer of the centrifuged suspension was pipetted (500 μl) into a new 1.5 ml Eppendorf tube. Afterward, 500 μl of isopropanol was added to the tube, after which the contents were mixed gently. The samples were pelleted by centrifugation at 12,000 × *g* (Rotor F45-24-11, Centrifuge 5415D, Eppendorf) for 5 min. The DNA pellet was subsequently washed with 70% ethanol and then dried in an oven for 1-2 min to remove ethanol. The DNA pellet was subsequently resuspended in 50 μl of sterilized distilled water and kept at -20 °C for further analysis.

### PCR detection

The detection process was carried out using specific primers (**Supplementary Table S1**). PCR was performed in a 40 μl reaction solution consisting of 100 ng of DNA, 40 ng of each forward and reverse primer, 0.05 mM dNTP, 0.25 U of *ProTaq* Plus DNA polymerase (Protech, Taipei, Taiwan) and reaction buffer. The conditions used for amplification were as follows: one cycle at 95°C for 5 min followed by 35 cycles of 95°C for 1 min, 50°C for 1 min and 72°C for 1 min. The PCR products were analyzed *via* electrophoresis involving a 0.8% agarose gel for 30 minutes with a voltage of 120 V.

### Construction of plasmid vectors, bimolecular fluorescence complementation and fluorescence resonance energy transfer analysis

Full-length begomoviral genes [the MP genes of ToLCNDV-OM and TYLCTHV, nuclear shuttle protein (NSP) gene of ToLCNDV-OM and ToLCNDV-CB, and CP gene of ToLCTV] were amplified with specific primers (**Supplementary Table S1**) by PCR with infectious DNA clones serving as templates. The PCR-amplified gene fragments were cloned and ligated into the Gateway entry vector pENTR/D-TOPO (Invitrogen, Thermo Fisher Scientific, Inc.) to generate pEN-OMMP, pEN-THMP, pEN-OMNSP, pEN-CBNSP, and pEN-TWCP vectors. The movement protein-coding genes of ToLCNDV-OM and TYLCTHV were then inserted into pK7WG2.0-N-YFP and pUBC-nYFP-Dest (Karimi et al., 2003) to generate pK2-YFPOMMP, pK2-YFPTHMP, pUBOMMPnYFP and pUBTHMPnYFP vectors. The NSP gene of ToLCNDV and the CP gene of ToLCTV were inserted into pK7WG2.0-N-CFP and pUBC-cYFP-Dest to generate pK2-CFPOMNSP, pK2-CFPCBNSP, pK2-CFPTWCP, pUBOMNSPcYFP, pUBCBNSPcYFP and pUBTWCPcYFP vectors. These constructs were individually transformed into the *Agrobacterium tumefaciens* strain C58 and coinfiltrated into *N. benthamiana* leaves for transient overexpression. Visualization of fluorophores and BiFC and FRET analysis were performed using an Olympus FV3000 confocal microscope (Tokyo, Japan) with different wavelength channels, namely, CFP (excitation 405 nm/emission 460-500 nm), YFP (excitation 488 nm/emission 530-630 nm), and FRET (excitation 405 nm/emission 530-630 nm), and analyzed using FV31S-SW software (Olympus).

## Results

### ToLCNDV-OM complemented the mechanical transmissibility of ToLCNDV-CB in *Nicotiana benthamiana*, cucumber, and oriental melon plants

**Figure 1:**
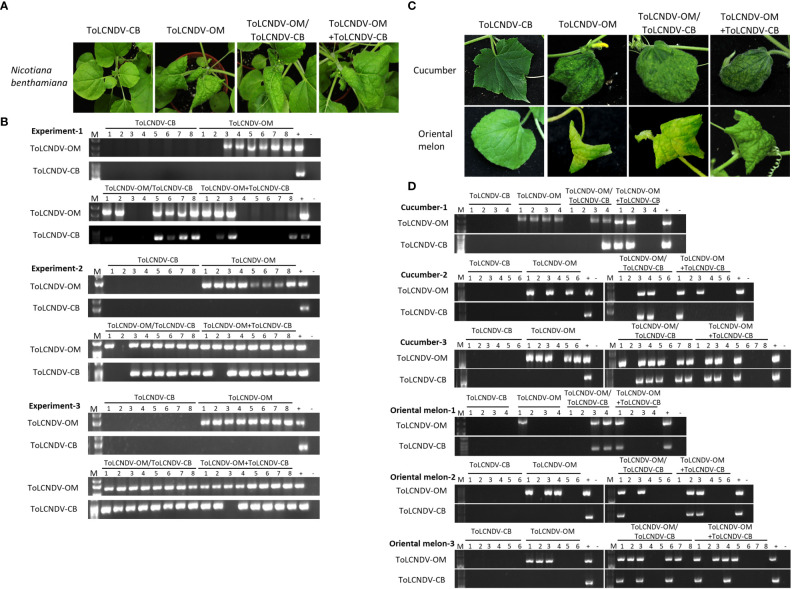
Symptom development and detection of tomato leaf curl New Delhi virus-oriental melon isolate (ToLCNDV-OM) and cucumber isolate (ToLCNDV-CB) in mechanically inoculated *Nicotiana benthamiana*, cucumber (*Cucumis sativus*) and oriental melon (*C. melo* var. *makuwa* cv. Silver Light). **(A)** Viruses in various combinations were mechanically inoculated into *N. benthamiana* plants. Severe leaf-curling symptoms were observed in the plants infected with ToLCNDV-OM, ToLCNDV-OM/ToLCNDV-CB, and ToLCNDV-OM+ToLCNDV-CB at 14 dpi. **(B)** Viruses in inoculated plants were detected *via* PCR with specific primers against DNA-B of ToLCNDV-OM and ToLCNDV-CB (**Supplementary Table S1**). Three biological replicates of *N. benthamiana* are individually presented. **(C)** Viruses in various combinations were mechanically inoculated into cucumber and oriental melon plants. Severe mosaic, yellowing, and leaf-curling symptoms were observed in plants infected with ToLCNDV-OM, ToLCNDV-OM/ToLCNDV-CB, and ToLCNDV-OM+ToLCNDV-CB at 14 dpi. **(D)** Viruses in inoculated plants were detected *via* PCR with specific primers against DNA-B of ToLCNDV-OM and ToLCNDV-CB (**Supplementary Table S1**). Three biological replicates of cucumber and oriental melon are individually presented. The positive control (+) for PCR detection is derived from plants infected with virus *via* agroinfiltration, and the negative control **(-)** is from uninoculated plants. The number indicates the number of plants used for inoculation.

ToLCNDV-OM and ToLCNDV-CB, which have different mechanical transmissibilities, are two different isolates of the same virus species. In our previous study, we documented that the mechanical transmissibility of ToLCNDV-OM and ToLCNDV-CB could be changed by the introduction of a specific amino acid mutation (Lee et al., 2020). Here, we tried to illustrate whether the mechanical transmissibility of ToLCNDV-CB can be complemented *via* coinfection with ToLCNDV-OM. Symptomatic leaves of agroinfiltrated plants were collected as inocula for mechanical inoculation of *N. benthamiana*. Severe leaf curl symptoms were observed for *N. benthamiana* mechanically inoculated with ToLCNDV-OM, ToLCNDV-OM/ToLCNDV-CB, and ToLCNDV-OM+ToLCNDV-CB at 14 dpi, while no symptoms were observed in *N. benthamiana* inoculated with ToLCNDV-CB alone (**Figure 1A**). The results of PCR detection with virus-specific primers showed that ToLCNDV-CB could be detected only in plants in the presence of ToLCNDV-OM (**Figure 1B**). Similar results were observed across three biological replicates. In the ToLCNDV-OM/ToLCNDV-CB and ToLCNDV-OM+ToLCNDV-CB experimental sets, the prevalence of ToLCNDV-CB was as high as 85% (17/20) in plants coinfected with ToLCNDV-OM (**Table 1**).

**Table 1:** Mechanical inoculation of tomato leaf curl New Delhi virus-oriental melon isolate (ToLCNDV-OM) and ToLCNDV-cucumber isolate (ToLCNDV-CB) on *Nicotiana benthamiana*, cucumber (*Cucumis sativus*) and oriental melon (*C. melo* var. *makuwa* cv. Silver Light).

Detection\Inoculation^†^	OM	CB	OM/CB	OM+CB
N. benthamiana^*^				
ToLCNDV-OM	22/24 (92%)^‡^	0/24 (0%)	21/24 (87.5%)	20/24 (83%)
ToLCNDV-CB	0/24 (0%) [0/22]^#^	0/24 (0%) [0/0]	19/24 (79.2%) [19/21]	17/24 (70.8) [17/20]
Cucumber^*^				
ToLCNDV-OM	12/16 (75%)	0/16 (0%)	10/18 (55.6%)	7/18 (38.9%)
ToLCNDV-CB	0/16 (0%) [0/12]	0/16 (0%) [0/0]	8/18 (44.4%) [8/10]	6/18 (33.3%) [6/7]
Oriental melon^*^				
ToLCNDV-OM	7/16 (43.8%)	0/16 (0%)	9/18 (50%)	7/18 (38.9%)
ToLCNDV-CB	0/16 (0%) [0/7]	0/16 (0%) [0/0]	6/18 (33.3%) [6/9]	5/18 (27.8%) [5/7]

^†^: Mechanical inoculation was conducted using inoculum derived from N. benthamiana that was either agroinfiltrated with ToLCNDV-OM (OM), or agroinfiltrated with ToLCNDV-CB (CB), or coagroinfiltrated with ToLCNDV-OM and ToLCNDV-CB (OM/CB) or inoculum with mixed saps from individually agroinfiltrated plants (OM+CB).

^‡^: Results of specific PCR detection were illustrated as positive plants/total inoculated plants (% infection rate).

^#^: Detection results were illustrated as coinfection ratio (CB positive plants/OM infected plants).

^*^: The results represent the combined data of three independent experiments.

To understand whether this phenomenon can also occur in cucurbit crop species, similar inoculations were performed on cucumber and oriental melon plants. Severe mosaic, yellowing, and leaf curl symptoms were observed in cucumber and oriental melon mechanically inoculated with ToLCNDV-OM, ToLCNDV-OM/ToLCNDV-CB, and ToLCNDV-OM+ToLCNDV-CB at 14 dpi, while no symptoms were observed in plants inoculated with ToLCNDV-CB alone (**Figure 1C**). Similar PCR results were observed, which showed that ToLCNDV-CB could be detected only in plants coinfected with ToLCNDV-OM (**Figure 1B**). Based on the results of the combined three replicates, infectivity of ToLCNDV-CB was as high as 80% (8/10) in cucumber and 66.7% (6/9) in oriental melon when both hosts were coinfected with ToLCNDV-OM (**Table 1**).

### TYLCTHV complemented the mechanical transmissibility of ToLCTV in *N benthamiana* and tomato plants

**Figure 2:**
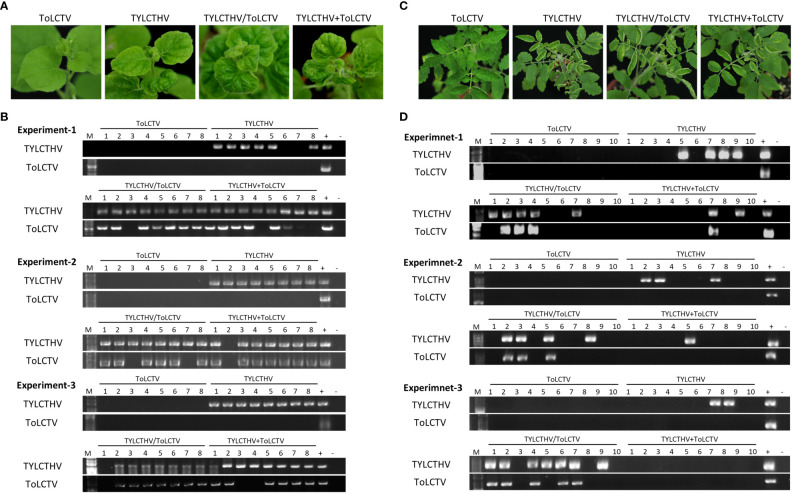
Symptom development and detection of tomato yellow leaf curl Thailand virus (TYLCTHV) and tomato leaf curl Taiwan virus (ToLCTV) in mechanically inoculated *Nicotiana benthamiana* and tomato (*Solanum lycopersicum* cv. ANT22). **(A)** Viruses in various combinations were mechanically inoculated in *N. benthamiana* plants. Severe mosaic and leaf curl symptoms were observed in plants infected with TYLCTHV, TYLCTHV/ToLCTV, and TYLCTHV+ToLCTV at 14 dpi. **(B)** Viruses in inoculated plants were detected *via* PCR with specific primers against DNA-B of TYLCTHV and DNA-A of ToLCTV (**Supplementary Table S1**). Three biological replicates of *N. benthamiana* are individually presented. **(C)** Viruses in various combinations were mechanically inoculated into cucumber and oriental melon plants. Severe mosaic and leaf curl symptoms were observed in plants infected with TYLCTHV, TYLCTHV/ToLCTV, and TYLCTHV+ToLCTV at 28 dpi. **(D)** Viruses in inoculated plants were detected *via* PCR with specific primers against DNA-B of TYLCTHV and DNA-A of ToLCTV (**Supplementary Table S1**). Three biological replicates of tomato are individually presented. The positive control (+) for PCR detection was derived from plants infected with virus *via* agroinfiltration, whereas the negative control **(-)** is from uninoculated plants. The number represents the number of plants used for inoculation.

To understand whether this complementation can occur between different viral species, TYLCTHV (a bipartite mechanically transmissible begomovirus) and ToLCTV (a monopartite nonmechanically transmissible begomovirus) were used for mechanical transmission analyses. Severe mosaic and leaf curl symptoms were observed in *N. benthamiana* mechanically inoculated with TYLCTHV, TYLCTHV/ToLCTV, and TYLCTHV+ToLCTV at 14 dpi, whereas no symptoms developed in *N. benthamiana* inoculated with ToLCTV alone (**Figure 2A**). The results of PCR detection showed that ToLCTV was detected only in plants coinfected with TYLCTHV (**Figure 2B**). For both the TYLCTHV/ToLCTV and TYLCTHV+ToLCTV inoculation methods, the prevalence of ToLCTV was as high as 86.9% (20/23) when the plants were coinfected with TYLCTHV (**Table 2**). The complementation analyses were also applied to tomato plants. Severe mosaic and leaf curl symptoms were observed at 28 dpi in tomato that were mechanically inoculated with TYLCTHV, TYLCTHV/ToLCTV, and TYLCTHV+ToLCTV but not with ToLCTV alone (**Figure 2C**). The PCR results also corroborated that, when coinfected with TYLCTHV, ToLCTV was mechanically transmitted only at 33.3% (1/3) (**Table 2**).

**Table 2:** Mechanical inoculation of tomato yellow leaf curl Thailand virus (TYLCTHV) and tomato leaf curl Taiwan virus (ToLCTV) on *Nicotiana benthamiana* and tomato (*Solanum lycopersicum* cv. ANT22).

Detection\Inoculation^†^	TH	TW	TH/TW	TH+TW
N. benthamiana^*^				
TYLCTHV	22/24 (91.7%)^‡^	0/24 (0%)	23/24 (95.8%)	23/24 (95.8%)
ToLCTV	0/24 (0%) [0/22]^#^	0/24 (0%) [0/0]	20/24 (83.3%) [20/23]	20/24 (83.3%) [20/23]
Tomato^*^				
TYLCTHV	9/30 (30%)	0/30 (0%)	16/30 (53.3%)	3/30 (10%)
ToLCTV	0/30 (0%) [0/9]	0/30 (0%) [0/0]	11/30 (36.7%) [11/16]	1/30 (3.3%) [1/3]

^†^: Mechanical inoculation on N. benthamiana and tomato was conducted using inoculum derived from plants either agroinfiltrated with TYLCTHV (TH), or agroinfiltrated with ToLCTV (TW), or coagroinfiltrated with TYLCTHV and ToLCTV (TH/TW), or inoculum with mixed saps from individually agroinfiltrated plants (TH+TW).

^‡^: Results of specific PCR detection were illustrated as positive plants/total inoculated plants (% infection rate).

^#^: Detection results were illustrated as coinfection ratio (TW positive plants/TH infected plants).

^*^: The results represented combined data of three independent experiments.

### Mechanically transmissible viruses altered the mechanical transmissibility and host range of other coinfecting viruses

**Figure 3:**
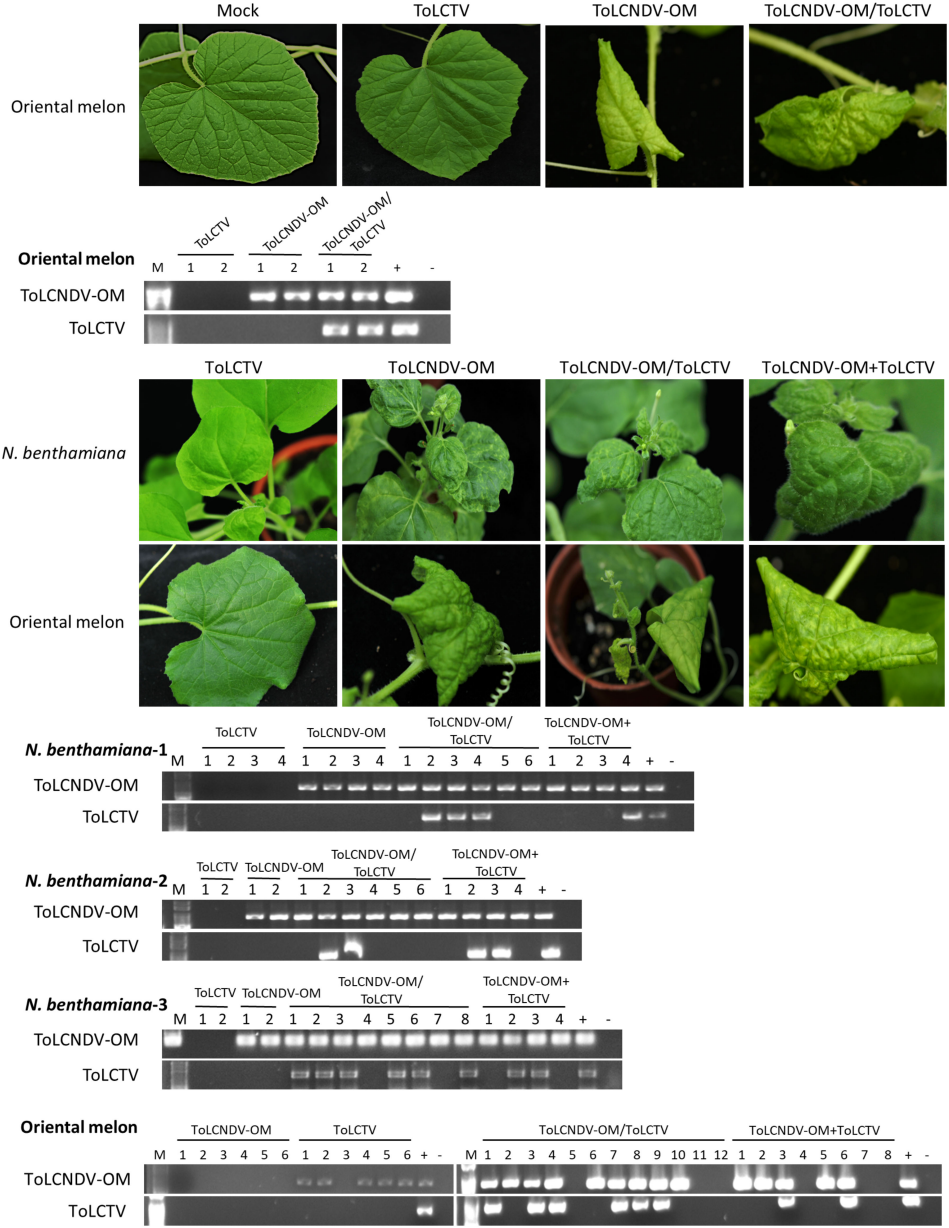
Symptom development and detection of tomato leaf curl New Delhi virus-oriental melon isolate (ToLCNDV-OM) and tomato leaf curl Taiwan virus (ToLCTV) in inoculated *Nicotiana benthamiana* and oriental melon (*C. melo* var. *makuwa* cv. Silver Light). **(A)** Viruses in various combinations were inoculated in oriental melon *via* agroinfiltration. Buffer infiltration was used as a mock control. **(B)** Viruses in agro-inoculated plants were detected *via* PCR with specific primers against DNA-B of ToLCNDV-OM and DNA-A of ToLCTV (**Supplementary Table S1**). **(C)** Viruses in various combinations were mechanically inoculated into *N. benthamiana* and oriental melon plants. Severe leaf curl symptoms were observed in *N. benthamiana* inoculated with ToLCNDV-OM, ToLCNDV-OM/ToLCTV and ToLCNDV-OM+ToLCTV, and severe mosaic patterns, yellowing, and leaf curling of oriental melon were observed at 14 dpi. **(D)** Viruses on inoculated plants were detected *via* PCR with specific primers against DNA-B of ToLCNDV-OM and DNA-A of ToLCTV (**Supplementary Table S1**). Three biological replicates of *N. benthamiana* are indicated, represented as *N. benthamiana*-1, 2, and 3. The results of the detection of all three biological replicates of oriental melon are presented together.

ToLCNDV-OM and ToLCNDV-CB were isolated from cucurbit plants, while TYLCTHV and ToLCTV were isolated from tomato plants. The results of agroinfiltration of ToLCTV alone showed that oriental melon was not a host of ToLCTV (**Figure 3A**). However, when coagroinfiltrated with ToLCNDV-OM, ToLCTV could be infectious (**Figures 3A, B**). Based on host adaptation, a virus might alter the cellular environment by creating an optimal habitat to enable a second virus to infect a non-host plant. To understand whether an adapted virus can facilitate the infection of a nonhost virus when both are coinoculated, mixed-infection experiments were conducted.

To understand whether this type of complementation can occur between viruses adapted to different hosts, tests were conducted involving the inoculation of ToLCNDV-OM with ToLCTV or TYLCTHV with ToLCNDV-CB. ToLCNDV-OM and ToLCTV were mechanically inoculated onto *N. benthamiana* and oriental melon plants in the abovementioned combinations. The *N. benthamiana* plants inoculated with ToLCNDV-OM/ToLCTV and ToLCNDV-OM+ToLCTV presented severe leaf curling, and the oriental melon plants presented severe mosaic signs, yellowing, and leaf curling at 14 dpi, the effects of which were similar to those induced by ToLCNDV-OM alone (**Figure 3C**). When coinoculated with ToLCNDV-OM+ToLCTV, ToLCTV could be detected in plants coinfected with ToLCNDV-OM (**Figure 3D**) at 40% (2/5) in oriental melon (**Table 3**). These results showed that ToLCTV was mechanically transmitted with ToLCNDV-OM to a nonhost oriental melon without affecting the symptoms induced by ToLCNDV-OM alone.

**Table 3:** Mechanical inoculation of tomato leaf curl New Delhi virus-oriental melon isolate (ToLCNDV-OM) and tomato leaf curl Taiwan virus (ToLCTV) on *Nicotiana benthamiana* and oriental melon (*C. melo* var. *makuwa* cv. Silver Light) plants.

Detection\Inoculation^†^	OM	TW	OM/TW	OM+TW
N. benthamiana^*^				
ToLCNDV-OM	8/8 (100%)^‡^	0/8 (0%)	20/20 (100%)	12/12 (100%)
ToLCTV	0/8 (0%) [0/8]^#^	0/8 (0%) [0/0]	11/20 (55%) [11/20]	5/12 (41.7%) [5/12]
Oriental melon^*^				
ToLCNDV-OM	5/6 (83.3%)	0/6 (0%)	9/12 (75%)	5/8 (62.5%)
ToLCTV	0/6 (0%) [0/5]	0/6 (0%) [0/0]	6/12 (50%) [6/9]	2/8 (25%) [2/5]

^†^: Mechanical inoculation on N. benthamiana and oriental melon was conducted using inoculum derived either from agroinfiltrated with ToLCNDV-OM (OM), or agroinfiltrated with ToLCTV (TW), or coagroinfiltrated with ToLCNDV-OM and ToLCTV (OM/TW), or inoculum with mixed saps from individually agroinfiltrated plants (OM+TW).

^‡^: Results of specific PCR detection were illustrated as positive plants/total inoculated plants (% infection rate).

^#^: Detection results were illustrated as coinfection ratio (TW positive plants/OM infected plants).

^*^: The results represented combined data of three independent experiments.

**Figure 4:**
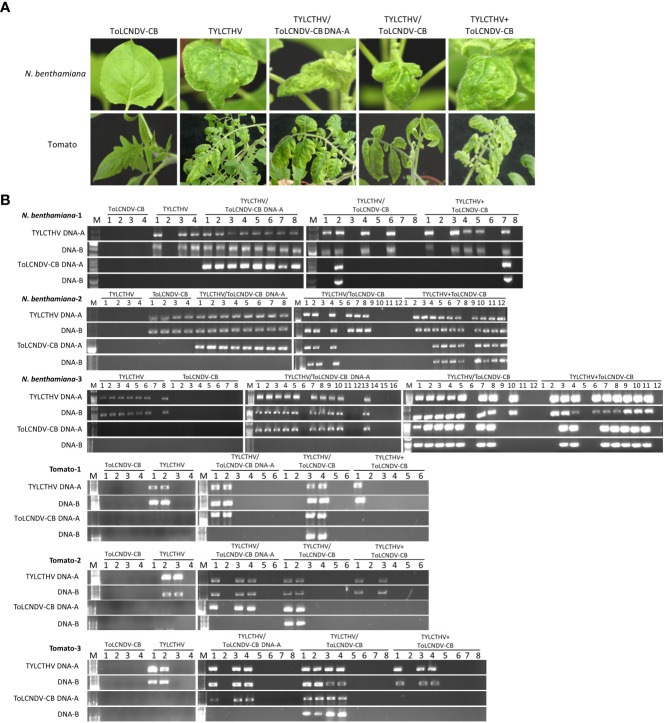
Symptom development and detection of tomato yellow leaf curl Thailand virus (TYLCTHV) and tomato leaf curl New Delhi virus-cucumber isolate (ToLCNDV-CB) on mechanically inoculated *Nicotiana benthamiana* and tomato (*Solanum lycopersicum* cv. ANT22). **(A)** Viruses in various combinations were mechanically inoculated into *N. benthamiana* and tomato plants. Severe mosaic and leaf curl symptoms were observed in *N. benthamiana* inoculated with TYLCTHV, TYLCTHV/ToLCNDV-CB, and TYLCTHV+ToLCNDV-CB at 14 dpi and in tomato at 28 dpi. **(B)** Viruses in inoculated plants were detected *via* PCR with specific primers against DNA-A and DNA-B of TYLCTHV and those of ToLCNDV-CB (**Supplementary Table S1**). Three biological replicates of *N. benthamiana* and tomato are individually presented.

*N. benthamiana* plants and tomato plants inoculated with TYLCTHV/ToLCNDV-CB and TYLCTHV+ToLCNDV-CB developed severe mosaic signs and leaf curling at 14 dpi and 28 dpi, respectively, the effects of which were similar to those induced by TYLCTHV alone (**Figure 4A**). ToLCNDV-CB could be detected in TYLCTHV/ToLCNDV-CB-inoculated *N. benthamiana* plants coinfected with TYLCTHV (**Figure 4B**) at 61.1% (11/18) (**Table 4**). This same test was also conducted by the inoculation of ToLCNDV-CB DNA-A with TYLCTHV on *N. benthamiana* and tomato. The infected plants developed symptoms similar to those of plants infected by TYLCTHV alone (**Figure 4A**). The PCR results showed that DNA-A of ToLCNDV-CB can be mechanically transmitted with TYLCTHV. These results showed that either DNA-A of ToLCNDV-CB alone or a complete ToLCNDV-CB could be mechanically transmitted with TYLCTHV to a nonhost tomato plants without altering symptoms induced by TYLCTHV alone.

**Table 4:** Mechanical inoculation of tomato yellow leaf curl Thailand virus (TYLCTHV) and tomato leaf curl New Delhi virus-cucumber isolate (ToLCNDV-CB) on *Nicotiana benthamiana* and tomato (*Solanum lycopersicum* cv. ANT22) plants.

Detection\Inoculation^†^	TH	CB	TH/CB-A	TH/CB	TH+CB
N. benthamiana^*^					
TYLCTHV	14/16 (87.5%)^‡^	0/16 (0%)	26/32 (81.25%)	18/32 (56.25%)	24/32 (75%)
ToLCNDV-CB	0/16 (0%) [0/14]^#^	0/16 (0%) [0/0]	26/32 (81.25%) [26/26]	11/32 (34.37%) [11/18]	16/32 (50%) [16/24]
Tomato^*^					
TYLCTHV	6/12 (50%)	0/12 (0%)	8/20 (40%)	8/20 (40%)	6/20 (30%)
ToLCNDV-CB	0/12 (0%) [0/6]	0/12 (0%) [0/0]	8/20 (40%) [8/8]	8/20 (40%) [8/8]	0/20 (0%) [0/6]

^†^: Mechanical inoculation on N. benthamiana and tomato was conducted using inoculum derived either from agroinfiltrated with TYLCTHV (TH), or agroinfiltrated with ToLCNDV-CB (CB), or coagroinfiltrated with TYLCTHV and ToLCNDV-CB DNA-A (TH/CB-A), or coagroinfiltrated with TYLCTHV and ToLCNDV-CB (TH/CB), or inoculum with mixed saps from individually agroinfiltrated plants (TH+CB).

^‡^: Results of specific PCR detection were illustrated as positive plants/total inoculated plants (% infection rate).

^#^: Detection results were illustrated as coinfection ratio (CB positive plants/TH infected plants).

^*^: The results represented combined data of three independent experiments.

### Existing mechanically transmissible viruses allowed the mechanical infection of secondary nonmechanically transmissible viruses

Viral infection in the field does not always occur in accordance with a coinfection manner simultaneously. In the present study, sequential inoculation was conducted with a mechanically transmissible virus for the first inoculation followed by another virus for the second inoculation. ToLCNDV-OM and TYLCTHV were first agroinfiltrated individually into *N. benthamiana* plants, which developed severe leaf curl symptoms two weeks later. The systemically symptomatic leaves infected with ToLCNDV-OM were then mechanically inoculated with ToLCNDV-CB, ToLCTV or TYLCTHV, while the plants infected with TYLCTHV were subsequently mechanically-inoculated with ToLCNDV-CB, ToLCTV, or ToLCNDV-OM. Two weeks later, the newest leaves from plants that received the 2^nd^ inoculation were collected for PCR-based detection. The results of PCR detection showed that TYLCTHV could be mechanically transmitted to plants infected with ToLCNDV-OM, with a 26.9% infection rate. However, ToLCNDV-OM could not be inoculated into plants infected with TYLCTHV. ToLCNDV-CB was detected in plants infected with ToLCNDV-OM and TYLCTHV and presented 53.8% and 69.2% infection rates, respectively. ToLCTV was detected in plants infected with TYLCTHV, with a 73.1% infection rate, and in plants infected with ToLCNDV-OM, with a 15.4% infection rate (**Table 5**). At sampling time, the plants exhibited severe mosaic and leaf curl symptoms; however, no obvious differences in symptoms were observed after secondary inoculations of various viruses were performed.

**Table 5:** Sequential mechanical inoculation of tomato yellow leaf curl Thailand virus (TYLCTHV), tomato leaf curl Taiwan virus (ToLCTV), tomato leaf curl New Delhi virus-cucumber isolate (ToLCNDV-CB), and oriental melon isolate (ToLCNDV-OM) on *Nicotiana benthamiana*.

1^st^ Inoculation^†^	TH			OM		
2^nd^ Inoculation^‡^ Detection\	OM	TW	CB	TH	TW	CB
TYLCTHV	26/26^*^	26/26	26/26	7/26	0/26	0/26
	(100%)	(100%)	(100%)	(26.9%)	(0%)	(0%)
ToLCNDV-OM	0/26	0/26	0/26	26/26	26/26	26/26
	(0%)	(0%)	(0%)	(100%)	(100%)	(100%)
ToLCNDV-CB	0/26	0/26	18/26	0/26	0/26	14/26
	(0%)	(0%)	(69.2%)	(0%)	(0%)	(53.8%)
ToLCTV	0/26	19/26	0/26	0/26	4/26	0/26
	(0%)	(73.1%)	(0%)	(0%)	(15.4%)	(0%)

The results are the combined data of three independent experiments.

^†^: 1^st^ inoculation was conducted by agroinfiltration of TYLCTHV (TH) and ToLCNDV-OM (OM)

^‡^: 2^nd^ inoculation was conducted mechanically. Mechanical inoculation on N. benthamiana was conducted using inoculum derived from either agroinfiltrated with TYLCTHV (TH), or agroinfiltrated with ToLCNDV-OM (OM), or agroinfiltrated with ToLCNDV-CB (CB), or agroinfiltrated with ToLCTV (TW). The second inoculation was conducted after plants exhibited symptoms from 1^st^ inoculation.

^*^: Results of specific PCR detection were illustrated as positive plants/total inoculated plants (% infection rate).

### Begomoviral MPs interacted with other viral proteins derived from different viruses

**Figure 5:**
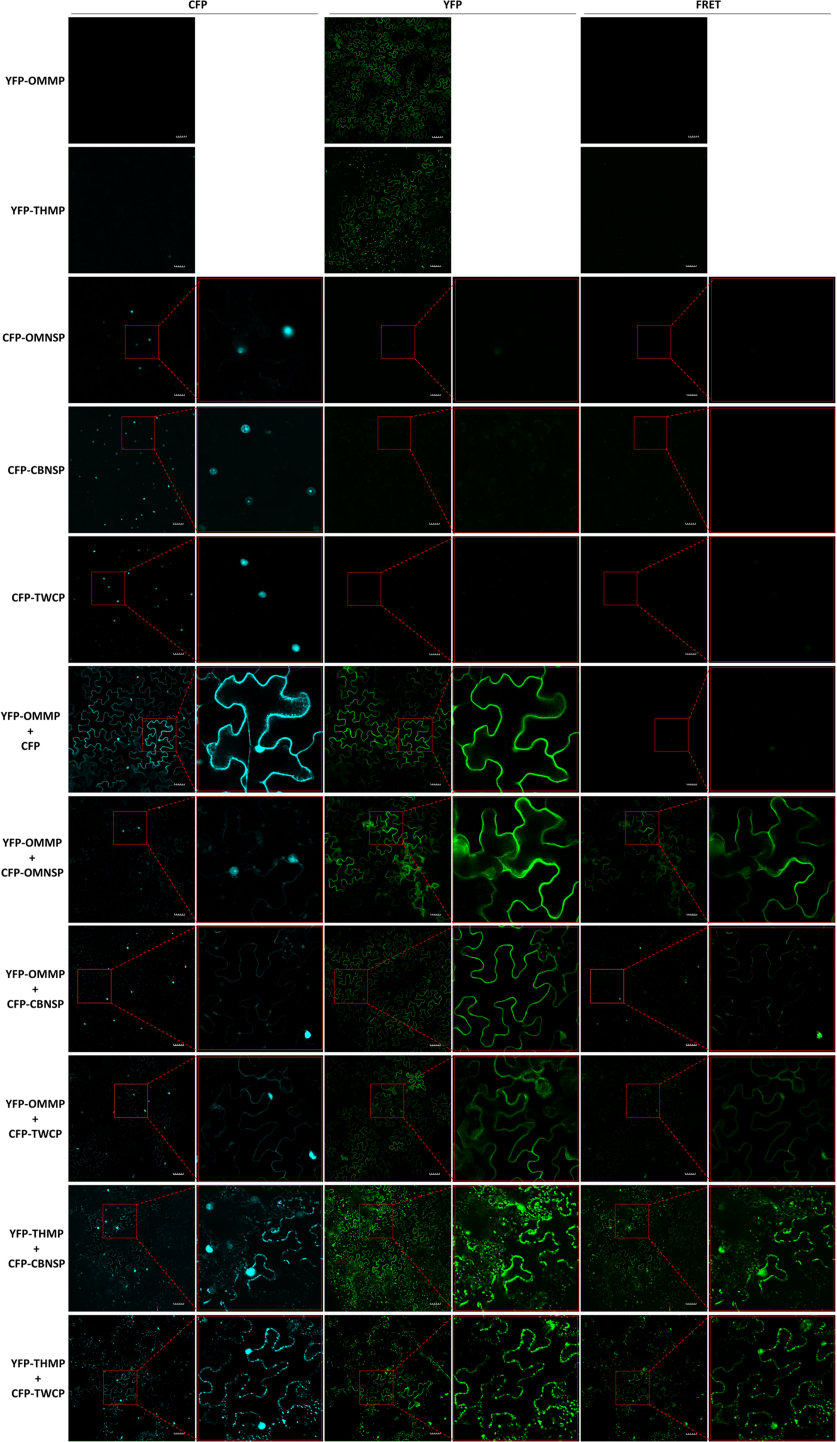
Fluorescence resonance energy transfer (FRET) analyses of *in planta* interactions between viral proteins derived from different begomoviruses. Movement protein-coding genes of tomato leaf curl New Delhi virus-oriental melon isolate (OMMP) and tomato yellow leaf curl Thailand virus (THMP) were fused to a yellow fluorescent protein (YFP) gene sequence, yielding pK2-YFPOMMP (YFP-OMMP) and pK2-YFPTHMP (YFP-THMP), respectively, and the nuclear shuttle protein-coding gene of ToLCNDV-oriental melon isolate (OMNSP) and ToLCNDV-cucumber isolate (CBNSP) and the coat protein gene of tomato leaf curl Taiwan virus (TWCP) were fused to a cyan fluorescent protein (CFP)-coding gene sequence, yielding pK2-CFPOMNSP (CFP-OMNSP), pK2-CFPCBNSP (CFP-CBNSP) and pK2-CFPTWCP (CFP-TWCP), respectively. These constructs were then expressed in leaf cells of *Nicotiana benthamiana via Agrobacterium*-mediated transformation. Agrobacteria carrying YFP-OMMP, YFP-THMP, CFP-OMNSP, CFP-CBNSP, and CFP-TWCP were infiltrated alone or coinfiltrated into *N. benthamiana* leaves. At two days post-infiltration, the leaves were collected and analyzed using an Olympus FV3000 confocal microscope at different wavelengths for detection of CFP (excitation 405 nm/emission 460-500 nm), YFP (excitation 488 nm/emission 530-630 nm), and FRET (excitation 405 nm/emission 530-630 nm). The resulting images were processed using FV31S-SW software. Scale bar = 50 µm. YFP-OMMP and CFP-OMNSP interactions (YFP-OMMP + CFP-OMNSP) were used as positive controls, and YFP-OMMP and CFP interactions (YFP-OMMP + CFP) were used as negative controls.

To further verify interactions between viral proteins derived from different begomoviruses, fluorescence resonance energy transfer (FRET) analyses were conducted with fusion proteins expressed in *N. benthamiana*. The yellow fluorescent protein (YFP) sequence was fused to the N-terminus of the MPs of ToLCNDV-OM (YFP-OMMP) and TYLCTHV (YFP-THMP). Yellow fluorescence (excitation 488 nm/emission 530-630 nm) emitted from the YFP-OMMP fusion protein was observed in the cells, and the fluorescence signal was evenly distributed along the periphery of the cells (**Figure 5**). A fluorescence signal of YFP-THMP was also observed, and the fluorescence formed spots along the periphery of the cells (**Figure 5**). The cyan fluorescent protein (CFP) sequence was fused to the N-terminus of the NSP-coding gene of ToLCNDV-OM (CFP-OMNSP) and ToLCNDV-CB (CFP-CBNSP) and the CP gene of ToLCTV (CFP-TWCP). Cyan fluorescence (excitation 405 nm/emission 460-500 nm) emitted from the CFP-OMNSP, CFP-CBNSP and CFP-TWCP fusion proteins was observed only in the nucleus (**Figure 5**). Interactions between YFP-OMMP and CFP-OMNSP were used as positive controls, while YFP-OMMP and CFP were used as negative controls. When YFP-OMMP was coexpressed with CFP-OMNSP, CFP-CBNSP or CFP-TWCP, the CFP signals were observed mainly in the nucleus, and some fluorescent signals formed continuous spots evenly displayed along the periphery of the cells (**Figure 5**). The FRET signals of YFP-OMMP/CFP-CBNSP and YFP-OMMP/CFP-TWCP were similar to those of the CFP signals (**Figure 5**). When YFP-THMP was coexpressed with CFP-CBNSP or CFP-TWCP, the CFP signals were observed mainly in the nucleus, and some formed spots along the periphery of the cells, similar to what YFP-THMP alone did (**Figure 5**). The FRET signals of YFP-OMMP/CFP-CBNSP and YFP-OMMP/CFP-TWCP were similar to those of the CFP signals (**Figure 5**). These results showed that the locations of CBNSP and TWCP were changed by OMMP or THMP and that OMMP/THMP interacted with CBNSP and TWCP along the periphery of the cells.

**Figure 6:**
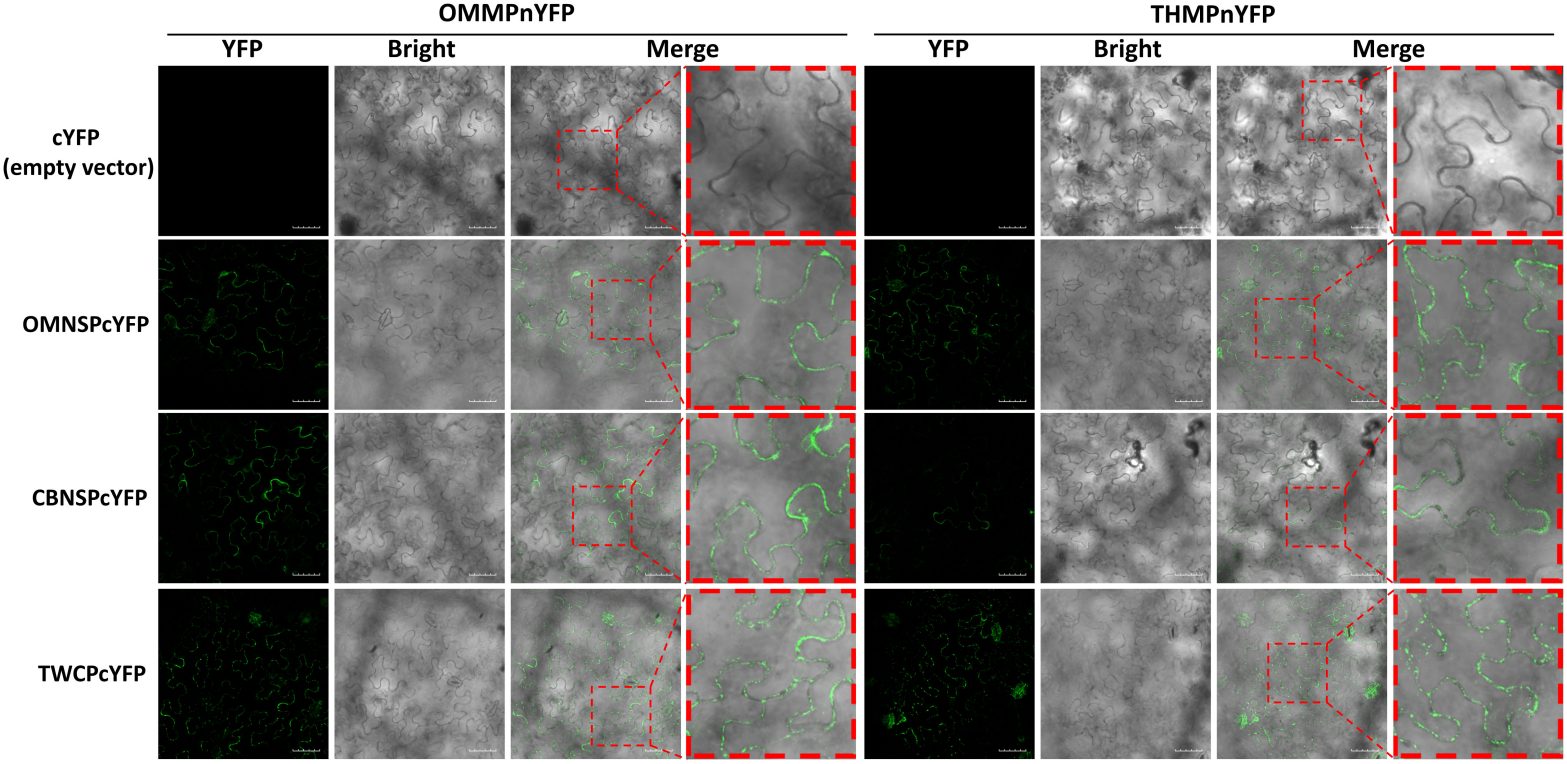
Bimolecular fluorescence complementation (BiFC) analyses of *in planta* interactions between viral proteins derived from different begomoviruses. Movement protein-coding genes of tomato leaf curl New Delhi virus-oriental melon isolate (OMMP) and tomato yellow leaf curl Thailand virus (THMP) were fused to the N half of the yellow fluorescent protein (YFP) gene sequence, yielding pUBOMMPnYFP (OMMPnYFP) and pUBTHMPnYFP (THMPnYFP), respectively. The nuclear shuttle protein-coding gene of ToLCNDV-oriental melon isolate (OMNSP) and ToLCNDV-cucumber isolate (CBNSP) and the coat protein gene of tomato leaf curl Taiwan virus (TWCP) were fused to C half of the YFP-coding gene sequence, yielding pUBOMNSPcYFP (OMNSPcYFP), pUBCBNSPcYFP (CBNSPcYFP) and pUBTWCPcYFP (TWCPcYFP), respectively. Agrobacteria carrying OMMPnYFP, THMPnYFP, OMNSPcYFP, CBNSPcYFP, and TWCPcYFP were infiltrated into *N. benthamiana* leaves. At two days post-infiltration, the leaves were collected and analyzed using an Olympus FV3000 confocal microscope for detection of YFP (excitation 488 nm/emission 530-630 nm) signal. Scale bar = 50 µm.

The protein-protein interactions were also analyzed with BiFC. The N half of YFP sequence was fused to the C-terminus of the MPs of ToLCNDV-OM and TYLCTHV to yield OMMPnYFP and OMMPnYFP, respectively. The C half of YFP sequence was fused to the C-terminus of the NSP-coding gene of ToLCNDV-OM (OMNSPcYFP) and ToLCNDV-CB (CBNSPcYFP) and the CP gene of ToLCTV (TWCPcYFP). Interactions between OMMPnYFP and OMNSPcYFP were used as positive controls, and OMMPnYFP and cYFP empty vector (cYFP) were used as negative controls. The YFP fluorescence was observed in different combinations, OMMPnYFP/CBNSPcYFP, OMMPnYFP/TWCPcYFP, THMPnYFP/CBNSPcYFP, and THMPnYFP/TWCPcYFP, mainly along the periphery of the cells, but not in nucleus (**Figure 6**). Taken together, these results indicated that MPs of bipartite begomoviruses, the determinants of mechanical transmissibility, interacted with movement-related proteins of other begomoviruses.

## Discussion

Infections involving a mixture of viruses can potentially trigger complex virus-virus interactions. Except for recombination, virus-virus interactions modify the characteristics of the infecting viruses, such as tissue tropism, infection and movement facilitation and insect transmissibility (Bourdin and Lecoq, 1991; Wege et al., 2001; Takács et al., 2014; Mascia and Gallitelli, 2016). Mechanical transmissibility is a critical factor for the spread of viruses in the field, which in turn makes disease management more complicated and difficult than simply controlling insect vectors alone. The mechanical transmissibility of begomoviruses is mainly determined by viral MPs (Lee et al., 2020), which always interact with viral DNA for intra- and intercellular trafficking. In this study, we found that nonmechanically transmissible begomoviruses can be complemented by a mechanically transmissible virus in a coinfection manner.

ToLCNDV-CB was mechanically transmitted to *N. benthamiana* and other crop species together with ToLCNDV-OM or TYLCTHV through coinoculation, as was ToLCTV. Based on our results, this complementation was observed in experiments involving coinoculation with both “virus A/virus B” and “virus A+virus B”. PCR detection and sequence analysis were used to confirm the presence of the infection viruses and rule out the possibility of contamination. These results corroborated that this complementation of mechanical transmissibility was not due to gene mutation or genomic recombination between the coinoculated viruses. In this study, the symptoms of the coinfected plants were not more severe than those of the single virus-infected plants (**Figures 1**-**4**). The accumulation of each virus detected by real-time PCR did not show consistent and significant changes (data not shown). These results revealed that the phenomena observed in this study were kinds of direct/indirect virus-virus interactions and not synergistic effects. Wege and Pohl (2007) reported that the DNA-B component supports the mechanical transmissibility of Abutilon mosaic virus (AbMV). Furthermore, our previous study indicated that the MP encoded in DNA-B is the key viral protein for mechanical transmissibility (Lee et al., 2020). ToLCNDV-OM and ToLCNDV-CB belong to the same virus species and have high nucleotide similarity (more than 92%) in terms of their genomic DNAs. The viral MP can recognize the genomic DNAs of both ToLCNDV-OM and ToLCNDV-CB. ToLCTV, a monopartite begomovirus, lacks DNA-B encoding an MP and an NSP. The mechanism of mechanical transmission and the role of MPs in mechanical transmission are still unclear. However, MPs of begomoviruses were reportedly able to bind nonspecifically to both ss- and dsDNA (Rojas et al., 1998; Hehnle et al., 2004; Radhakrishnan et al., 2008). This may partly explain why ToLCTV can be mechanically transmitted with ToLCNDV-OM and TYLCTHV. Similarly, ToLCNDV-CB DNA-A alone can be complemented through mixed infection with TYLCTHV for systemic movement and mechanical transmission in *N. benthamiana* (**Table 4** and **Figure 4**). In other words, the DNA-B of a mechanically transmissible begomovirus encodes an MP for systemic infection and mechanical transmission. This MP may also help a coinfected nonmechanically transmissible begomovirus to be mechanically transmitted through an unclear virus-virus interaction, such as MP-viral protein, MP-viral DNA or MP-host-virus interaction.

The results of sequential inoculation showed that nonmechanically transmissible ToLCNDV-CB and ToLCTV could be inoculated *via* sap into plants infected with ToLCNDV-OM or TYLCTHV. Complementation of mechanical transmissibility occurred not only in coinoculation but also in a sequential inoculation manner. It is assumed that the mechanical transmissibility of begomoviruses is not only determined by viral movement but also affected by host status. The preexisting mechanically transmissible begomovirus may switch host resistance against or a pathway allowing mechanical inoculation of other infecting begomoviruses. Further analysis is needed to accurately understand this regulation. Interestingly, TYLCTHV and ToLCNDV-OM were used as positive controls of mechanical transmission for inoculation into plants preinfected with ToLCNDV-OM and TYLCTHV. However, the results showed that ToLCNDV-OM cannot be mechanically inoculated into plants infected with TYLCTHV, whereas TYLCTHV can infect plants preinfected with ToLCNDV-OM (**Table 5**). Nonetheless, the competition or antagonism induced by TYLCTHV against ToLCNDV-OM warrants further study.

Based on our inoculation analyses, the “virus A+virus B” experimental set in most combinations showed lower transmission rates than did the “virus A/virus B” set. At least two likely possibilities can explain why this occurred. First, to minimize the inhibitors derived from host tissues, the inoculum was prepared such that there was an equal ratio of tissue weight/buffer volume. The “virus A+virus B” set containing tissues from two plants resulted in a lower viral titer in the inoculum solution than that in the “virus A/virus B” set. Second, the lower transmission rates may be associated with the timing of virus-virus interactions. Klinkenberg et al. (1989) reported that the time of interaction between viral DNA and protein could affect complex formation. Inoculants originating from the “virus A/virus B” plants may have already engaged in earlier interactions between viral DNAs and viral proteins, whereas those interactions may not yet definitively occur for the inoculants originating from the “virus A+virus B” set.

Nonspecific binding of CP and viral RNA resulted in heteroencapsidation that altered a nonaphid-transmissible potyvirus to become aphid transmissible, as reported by Bourdin and Lecoq (1991). In this study, we discovered the complementation of mechanical transmissibility in coinfected begomoviruses, and we also tried to determine possible mechanisms involved in this complementation. Based on the functions of begomoviral MPs, which participate in virus movement and mechanical transmissibility, we hypothesized that complementation occurred through interactions between MPs and viral DNA or viral proteins. MPs and NSPs of several begomoviruses were reportedly bound to DNA in a nonspecific manner (Pascal et al., 1994; Rojas et al., 1998; Hehnle et al., 2004). The coat protein of monopartite geminiviruses plays roles similar to those played by NSPs of bipartite begomoviruses—shuttling viral DNAs between the nucleus and cytoplasm and being involved in spreading in hosts (Rigden et al., 1993; Sanderfoot et al., 1996; Liu et al., 2001; Rojas et al., 2001). The NSP of AbMV expressed alone localizes to the nucleus, while, expressed with MP, it localizes to both the nucleus and cellular periphery (Happle et al., 2021). When the CP of tomato yellow leaf curl virus (TYLCV) is expressed alone or is coexpressed with replication-associated proteins of TYLCV in host cells, it localizes mainly to the nucleus (Kunik et al., 1998; Wang et al., 2017). Although the subcellular localization of many geminiviral proteins has been analyzed, the colocalization and interaction of viral proteins derived from different geminiviruses is still unclear. In this study, the results of FRET analyses showed that the NSP of ToLCNDV-CB and the CP of ToLCTV localized to the nucleus when expressed alone (**Figure 5**). Although dynamic translocation needs further analysis, these two proteins coexpressed with MPs of ToLCNDV-OM or TYLCTHV relocalized to both the nucleus and cellular periphery and interacted with MPs (**Figure 5**). Taken together, these results show that begomoviral proteins involved in intra- and intercellular movement can nonspecifically interact with DNAs and movement proteins of another coinfecting begomovirus, and this interaction may lead to the comovement and cotransmission of mix-infecting begomoviruses.

Méndez-Lozano et al. (2003) reported the following two phenomena observed in coinfected pepper huasteco virus and pepper golden mosaic virus: functional complementation of comovement and heterologous transactivation of CP promoters. These findings indicated that a begomovirus can complement both the gene expression and protein function of another coinfecting begomovirus. The host range of ToLCTV is reportedly limited to tomato (*Solanum* spp.), *S. melongena*, *N. benthamiana*, *Datura stramonium*, *Lonicera japonica*, *Petunia hybrida*, and *Physalis floridana* (Green et al., 1987). In our host range test involving agroinfiltration, ToLCTV did not infect oriental melon (**Figures 3A, B**), and ToLCNDV-CB did not infect tomato (data not shown). In this study, the results showed that, as part of a mixed infection with ToLCNDV-OM, ToLCTV infected nonhost oriental melon through agroinfiltration and mechanical inoculation (**Figures 3A, C**). ToLCNDV-CB was also mechanically transmitted to nonhost tomato plants with TYLCTHV (**Figure 4A**). The mechanically transmissible virus complemented the mechanical transmissibility and altered the host range of another coinfection virus. Alteration of the host range not only demonstrated the scenario of MP-mediated transmissibility but also illustrated how a virus replicates, expresses, and moves in a nonhost plant. The first expressed replication-associated protein (Rep) of begomoviruses recognizes the replication origin in a virus-specific manner, and recruits the host DNA replication machinery to viral genome (Fontes et al., 1994; Fondong, 2013; Hanley-Bowdoin et al., 2013; Rizvi et al., 2015). The replication enhancer protein (REN) interacts with DNA polymerase subunits and plays an ancillary role in replication (Wu et al., 2021). An adapted begomovirus may turn on the host machinery for viral replication and benefit another coinfecting nonhost begomovirus. Transcription of begomoviral proteins relies nearly on the host machinery with a conserved RNA polymerase II system (Jeske, 2009; Hanley-Bowdoin et al., 2013). Early expressed transcriptional activator protein (TrAP) activates the transcription of CP and MP in a nonvirus-specific manner (Saunders and Stanley, 1995; Hanley-Bowdoin et al, 1999). Taking this information and the abovementioned nonspecific interactions of MP and CP for begomoviral movement and transmission together, mixed infections of begomoviruses can help each other in terms of propagation, movement, and transmission. This may explain why ToLCTV and ToLCNDV-CB infect nonhost plants in mixed-infection manners with ToLCNDV-OM and TYLCTHV. In addition, more physical interactions may also produce more virus recombinants, and more complicated virus interactions lead to more complicated epidemiology.

Based on a previous report by Tsai et al. (Tsai et al., 2011), throughout Taiwan in 2008-2009, TYLCTHV dominated the tomato planting area, with an incidence of 51%, while ToLCTV accounted for only 8%. This study demonstrated that the incidence caused by ToLCTV increased by 41% if this virus were mixed with TYLCTHV in the field (Tsai et al., 2011). Because of the use of greenhouses and insecticides in Taiwan, whitefly transmission has been reduced. Cutting, grafting, and thinning are common practices applied in the field that make mechanical transmission an even more efficient way for virus spreading. Overall, the single infection of ToLCTV that resulted in the lower distribution of the virus could well be attributed to its nonmechanical transmissibility. Once coinfection occurs, ToLCTV can be spread with TYLCTHV through mechanical transmission in the field. Indeed, most ToLCTVs were detected in coinfection samples.

In this study, we tried to analyze the complementation of mechanical transmissibility between begomovirus infection mixtures. Our results indicated that complex virus-virus interactions occurred during mixed infection and that these interactions complemented the mechanical transmissibility and altered the host range of the begomoviruses. This complementation can occur between unrelated and different species of either monopartite or bipartite begomoviruses. These findings indicated that the spread of begomoviruses *via* mechanical transmission is more likely than expected and consequently makes disease management more difficult. Complementation of mechanical transmissibility in sequential infection increased the rate of mixed infection in the field, and those complex interactions led to trait changes such as altered host range. Although our FRET and BiFC analyses revealed protein-protein interactions between different viral proteins, the virus-virus interaction leading to the alterations of mechanical transmission and host range may happen in other ways through an indirect or non-physical contact between begomoviruses. Further studies to elucidate virus-virus interactions will provide critical insights to aid in strategizing the control of viral spreading and reducing disease severity in the field.

## Data availability statement

The original contributions presented in the study are included in the article/**Supplementary Material**. Further inquiries can be directed to the corresponding author.

## Author contributions

H-HC designed the methods, performed the experiments, interpreted the data, and wrote the original draft. DG designed and performed the experiments, interpreted the data, and revised the manuscript. C-JC interpreted the data and revised the manuscript. F-JJ administered the research project, planned and designed the research, interpreted the data, and revised the manuscript. H-HC and DG contributed equally to this work. All authors contributed to the article and approved the submitted version.

## References

[B1] Alves-Júnior M. Alfenas-Zerbini P. Andrade E. C. Esposito D. A. Silva F. N. da Cruz A. C. F.. (2009). Synergism and negative interference during co-infection of tomato and nicotiana benthamiana with two bipartite begomoviruses. Virology 387, 257–266. doi: 10.1016/j.virol.2009.01.046 19282016

[B2] Bourdin D. Lecoq H. (1991). Evidence that heteroencapsidation between two potyviruses is involved in aphid transmission of a non-aphid-transmissible isolate from mixed infections. Phytopathology 81, 1459–1464. doi: 10.1094/Phyto-81-1459

[B3] Chakraborty S. Vanitharani R. Chattopadhyay B. Fauquet C. M. (2008). Supervirulent pseudorecombination and asymmetric synergism between genomic components of two distinct species of begomovirus associated with severe tomato leaf curl disease in India. J. Gen. Virol. 89, 818–828. doi: 10.1099/vir.0.82873-0 18272774

[B4] Chang H.-H. Ku H.-M. Tsai W.-S. Chien R.-C. Jan F.-J. (2010). Identification and characterization of a mechanical transmissible begomovirus causing leaf curl on oriental melon. Eur. J. Plant Pathol. 127, 219–228. doi: 10.1007/s10658-010-9586-0

[B5] Chang H.-H. Lee C.-H. Chang C.-J. Jan F.-J. (2022). FKBP-type peptidyl-propyl cis-trans isomerase interacts with the movement protein of tomato leaf curl new Delhi virus and impacts viral replication in *Nicotiana benthamiana* . Mol. Plant Pathol. 23, 561–575. doi: 10.1111/mpp.13181 34984809 PMC8916215

[B6] DaPalma T. Doonan B. P. Trager N. M. Kasman L. M. (2010). A systematic approach to virus-virus interactions. Virus Res. 149, 1–9. doi: 10.1016/j.virusres.2010.01.002 20093154 PMC7172858

[B7] Elena S. F. Bedhomme S. Carrasco P. Cuevas J. M. de la Iglesia F. Lafforgue G.. (2011). The evolutionary genetics of emerging plant RNA viruses. Mol. Plant-Microbe Interact. 24, 287–293. doi: 10.1094/MPMI-09-10-0214 21294624

[B8] Fiallo-Olivé E. Lett J.-M. Martin D. P. Roumagnac P. Varsani A. Zerbini F. M.. (2021). ICTV virus taxonomy profile: geminiviridae 2021. J. Gen. Virol. 102, 001696. doi: 10.1099/jgv.0.001696 34919512 PMC8744271

[B9] Folimonova S. Y. (2012). Superinfection exclusion is an active virus-controlled function that requires a specific viral protein. J. Virol. 86, 5554–5561. doi: 10.1128/JVI.00310-12 22398285 PMC3347309

[B10] Fondong V. N. (2013). Geminivirus protein structure and function. Mol. Plant Pathol. 14, 635–649. doi: 10.1111/mpp.12032 23615043 PMC6638828

[B11] Fontes E. P. B. Eagle P. A. Sipe P. S. Luckow V. A. Hanley-Bowdoin L. (1994). Interaction between a geminivirus replication protein and origin DNA is essential for viral replication. J. Biol. Chem. 269, 8459–8465. doi: 10.1016/S0021-9258(17)37216-2 8132573

[B12] Fukuzawa N. Itchoda N. Ishihara T. Goto K. Masuta C. Matsumura T. (2010). HC-pro, a potyvirus RNA silencing suppressor, cancels cycling of cucumber mosaic virus in *Nicotiana benthamiana* plants. Virus Genes 40, 440–446. doi: 10.1007/s11262-010-0460-0 20162445

[B13] García-Arenal F. Zerbini F. M. (2019). Life on the edge: geminiviruses at the interface between crops and wild plant hosts. Annu. Rev. Virol. 6, 411–433. doi: 10.1146/annurev-virology-092818-015536 31180812

[B14] Green S. K. Sulyo Y. Lesemann D. E. (1987). Outbreak and new records: leaf curl virus on tomato in Taiwan province. FAO Plant Prot Bull. 35, 62.

[B15] Hanley-Bowdoin L. Bejarano E. R. Robertson D. Mansoor S. (2013). Geminiviruses: masters at redirecting and reprogramming plant processes. Nat. Rev. Microbiol. 11, 777–788. doi: 10.1038/nrmicro3117 24100361

[B16] Hanley-Bowdoin L. Settlage S. B. Orozco B. M. Nagar S. Robertson D. (1999). Geminiviruses: models for plant DNA replication, transcription, and cell cycle regulation. Crit. Rev. Plant Sci. 18, 71–106. doi: 10.1080/07352689991309162 10821479

[B17] Happle A. Jeske H. Kleinow T. (2021). Dynamic subcellular distribution of begomoviral nuclear shuttle and movement proteins. Virology 562, 158–175. doi: 10.1016/j.virol.2021.07.014 34339930

[B18] Hehnle S. Wege C. Jeske H. (2004). The interaction of DNA with the movement proteins of geminiviruses revisited. Virology 78, 7698–7706. doi: 10.1128/JVI.78.14.7698-7706.2004 PMC434128 15220444

[B19] Jeske H. (2009). Geminiviruses. Curr. Top. Microbiol. Immunol. 331, 185–226. doi: 10.1007/978-3-540-70972-5_11 19230564

[B20] Kanakala S. Jyothsna P. Shukla R. Tiwari N. Veer B. S. Swarnalatha P.. (2013). Asymmetric synergism and heteroencapsidation between two bipartite begomoviruses, tomato leaf curl new Delhi virus and tomato leaf curl palampur virus. Virus Res. 174, 126–136. doi: 10.1016/j.virusres.2013.03.011 23578824

[B21] Karimi M. Inzé D. Depicker A. (2003). GATEWAYTM vectors for agrobacterium-mediated plant transformation. Trends Plant Sci. 7, 193–195. doi: 10.1016/S1360-1385(02)02251-3 11992820

[B22] Karyeija R. F. Kreuze J. F. Gibson R. W. Valkonen J. P. T. (2000). Synergistic interactions of a potyvirus and a phloem-limited crinivirus in sweet potato plants. Virology 269, 26–36. doi: 10.1006/viro.1999.0169 10725195

[B23] Klinkenberg F. A. Ellwood S. Stanley J. (1989). Fate of African cassava mosaic virus coat protein deletion mutants after agroinoculation. J. Gen. Virol. 70, 1837–1844. doi: 10.1099/0022-1317-70-7-1837 2732690

[B24] Kunik T. Palanichelvam K. Czosnek H. Citovsky V. Gafni Y. (1998). Nuclear import of the capsid protein of tomato yellow leaf curl virus (TYLCV) in plant and insect cells. Plant J. 13, 393–399. doi: 10.1046/j.1365-313X.1998.00037.x 9680988

[B25] Lee C.-H. Zheng Y.-X. Chan C.-H. Ku H.-M. Chang C.-J. Jan F.-J. (2020). A single amino acid substitution in the movement protein enables the mechanical transmission of a geminivirus. Mol. Plant Pathol. 21, 571–588. doi: 10.1111/mpp.12917 32078762 PMC7060137

[B26] Lefeuvre P. Moriones E. (2015). Recombination as a motor of host switches and virus emergence: geminiviruses as case studies. Curr. Opin. Virol. 10, 14–19. doi: 10.1016/j.coviro.2014.12.005 25559880

[B27] Lin C.-Y. Tsai W.-S. Ku H.-M. Jan F.-J. (2012). Evaluation of DNA fragments covering the entire genome of a monopartite begomovirus for induction of viral resistance in transgenic plants *via* gene silencing. Transgenic Res. 21, 231–241. doi: 10.1007/s11248-011-9523-9 21597979

[B28] Lin S.-S. Wu H.-W. Jan F.-J. Hou R. F. Yeh S.-D. (2007). Modifications of the helper component-protease of zucchini yellow mosaic virus for generation of attenuated mutants for cross protection against severe infection. Phytopathology 97, 287–296. doi: 10.1094/PHYTO-97-3-0287 18943647

[B29] Liu H. Lucy A. P. Davies J. Boulton M. I. (2001). A single amino acid change in the coat protein of maize streak virus abolishes systemic infection, but not interaction with viral DNA of movement protein. Mol. Plant Pathol. 2, 223–228. doi: 10.1046/j.1464-6722.2001.00068.x 20573010

[B30] Lu B. Stubbs G. Culver J. N. (1998). Coat protein interactions involved in tobacco mosaic tobamovirus cross-protection. Virology 248, 188–198. doi: 10.1006/viro.1998.9280 9721228

[B31] Mascia T. Gallitelli D. (2016). Synergies and antagonisms in virus interactions. Plant Sci. 252, 176–192. doi: 10.1016/j.plantsci.2016.07.015 27717453

[B32] Mckinney H. H. (1929). Mosaic diseases in the canary islands, West Africa and Gibraltar. J. Agric. Res. 39, 577–578. Available at: https://handle.nal.usda.gov/10113/IND43967734.

[B33] Méndez-Lozano J. Torres-Pacheco I. Fauquet C. M. Rivera-Bustamante R. F. (2003). Interactions between geminiviruses in a naturally occurring mixture: pepper huasteco virus and pepper golden mosaic virus. Phytopathology 93, 270–277. doi: 10.1094/PHYTO.2003.93.3.270 18944336

[B34] Nawaz-Ul-Rehman M. S. Fauquet C. M. (2009). Evolution of geminiviruses and their satellite. FEBS Lett. 583, 1825–1832. doi: 10.1016/j.febslet.2009.05.045 19497325

[B35] Oberemok V. Laikova K. Golovkin I. Kryukov L. Kamenetsky-Goldstein R. (2021). Biotechnology of virus eradication and plant vaccination in phytobiome context. Plant Biol. 24, 3–8. doi: 10.1111/plb.13338 34569131

[B36] Pascal E. Sanderfoot A. A. Ward B. M. Medville R. Turgeon R. Lazarowitz S. G. (1994). The geminivirus BR1 movement protein binds single-stranded DNA and localizes to the cell nucleus. Plant Cell 6, 995–1006. doi: 10.1105/tpc.6.7.995 8069108 PMC160495

[B37] Pechinger K. Chooi K. M. Macdiarmid R. M. Harper S. J. Ziebell H. (2019). A new era for mild strain cross-protection. Viruses 11, 670. doi: 10.3390/v11070670 31340444 PMC6669575

[B38] Radhakrishnan G. K. Splitter G. A. Usha R. (2008). DNA Recognition properties of the cell-to-cell movement protein (MP) of soybean isolate of mungbean yellow mosaic India virus (MYMIV-Sb). Virus Res. 131, 152–159. doi: 10.1016/j.virusres.2007.09.002 17949843

[B39] Redinbaugh M. G. Stewart L. R. (2018). Maize lethal necrosis: an emerging, synergistic viral disease. Annu. Rev. Virol. 5, 301–322. doi: 10.1146/annurev-virology-092917-043413 30059641

[B40] Rentería-Canett I. Xoconostle-Cázares B. Ruiz-Medrano R. Rivera-Bustamante R. F. (2011). Geminivirus mixed infection on pepper plants: synergistic interaction between PHYVV and PepGMV. Virol. J. 8, 104. doi: 10.1186/1743-422X-8-104 21385390 PMC3061938

[B41] Rigden J. E. Kdry I. B. Mullineaux P. M. Rezaian M. A. (1993). Mutagenesis of the virion-sense open reading frames of tomato leaf curl virus. Virology 193, 1001–1005. doi: 10.1006/viro.1993.1215 8460471

[B42] Rizvi I. Choudhury N. R. Tuteja N. (2015). Insights into the functional characteristics of geminivirus rolling-circle replication initiator protein and its interaction with host factors affecting viral DNA replication. Arch. Virol. 160, 375–387. doi: 10.1007/s00705-014-2297-7 25449306

[B43] Rojas M. R. Jiang H. Salati R. Xoconostle-Cazares B. Sudarshana M. R. Lucas W. J.. (2001). Functional analysis of proteins involved in movement of the monopartite begomovirus, tomato yellow leaf curl virus. Virology 291, 110–125. doi: 10.1006/viro.2001.1194 11878881

[B44] Rojas M. R. Noueiry A. O. Lucas W. J. Gilbertson R. L. (1998). Bean dwarf mosaic geminivirus movement proteins recognize DNA in a form- and size-specific manner. Cell 95, 105–113. doi: 10.1016/S0092-8674(00)81786-9 9778251

[B45] Sanderfoot A. A. Ingham D. J. Lazarowitz S. G. (1996). A viral movement protein as a nuclear shuttle. the geminivirus BR1 movement protein contains domains essential for interaction with BL1 and nuclear localization. Plant Physiol. 110, 23–33. doi: 10.1104/pp.110.1.23 8587985 PMC157690

[B46] Saunders K. Stanley J. (1995). Complementation of African cassava mosaic virus AC2 gene function in a mixed bipartite geminivirus infection. J. Gen. Virol. 76, 2287–2292. doi: 10.1099/0022-1317-76-9-2287 7561766

[B47] Sufrin-Ringwald T. Lapidot M. (2011). Characterization of a synergistic interaction between two cucurbit-infecting begomoviruses: squash leaf curl virus and watermelon chlorotic stunt virus. Phytopathology 101, 281–289. doi: 10.1094/PHYTO-06-10-0159 21219130

[B48] Syller J. (2012). Facilitative and antagonistic interactions between plant viruses in mixed infections. Mol. Plant Pathol. 13, 204–216. doi: 10.1111/j.1364-3703.2011.00734.x 21726401 PMC6638836

[B49] Sztuba-Solińska J. Urbanowicz A. Figlerowicz M. Bujarski J. J. (2011). RNA-RNA Recombination in plant virus replication and evolution. Annu. Rev. Phytopathol. 49, 415–443. doi: 10.1146/annurev-phyto-072910-095351 21529157

[B50] Takács A. Gáborjányi R. Horváth J. Kazinczi G. (2014). "Virus-virus interaction," in Plant virus-host interaction: Molecular approaches and viral evolution eds. Gaur R. K. Hohn T. Sharma P.. (MA, USA: Academic Press), 385–394. doi: 10.1016/B978-0-12-411584-2.00021-4

[B51] Tatineni S. Graybosch R. A. Hein G. L. Wegulo S. N. French R. (2010). Wheat cultivar-specific disease synergism and alteration of virus accumulation during co-infection with wheat streak mosaic virus and triticum mosaic virus. Phytopathology 100, 230–238. doi: 10.1094/PHYTO-100-3-0230 20128696

[B52] Tran T.-T.-Y. Lin T.-T. Chang C.-P. Chen C.-H. Nguyen V.-H. Yeh S.-D. (2022). Generation of mild recombinants of papaya ring spot virus to minimize the problem of strain-specific cross-protection. Phytopathology 112, 708–719. doi: 10.1094/PHYTO-06-21-0272-R 34384243

[B53] Tsai W.-S. Shih S.-L. Kenyon L. Green S. K. Jan F.-J. (2011). Temporal distribution and pathogenicity of the predominant tomato-infecting begomoviruses in Taiwan. Plant Pathol. 60, 787–799. doi: 10.1111/j.1365-3059.2011.02424.x

[B54] Wang L. Tan H. Wu M. Jimenez-Gongora T. Tan L. Lozano-Duran R. (2017). Dynamic virus-dependent subnuclear localization of the capsid protein form a geminivirus. Front. Plant Sci. 8. doi: 10.3389/fpls.2017.02165 PMC5744400 29312406

[B55] Wege C. Pohl D. (2007). Abutilon mosaic virus DNA b component supports mechanical virus transmission, but does not counteract begomoviral phloem limitation in transgenic plants. Virology 36, 173–186. doi: 10.1016/j.virol.2007.03.041 17462695

[B56] Wege C. Saunders K. Stanley J. Jeske H. (2001). Comparative analysis of tissue tropism of bipartite geminiviruses. J. Phytopathol. 149, 359–368. doi: 10.1046/j.1439-0434.2001.00640.x

[B57] Wu M. Wei H. Tan H. Pan S. Liu Q. Bejarano E. R.. (2021). Plant DNA polymerases α and δ mediate replication of geminiviruses. Nat. Commun. 12, 2780. doi: 10.1038/s41467-021-23013-2 33986276 PMC8119979

[B58] Xu Y. Ghanim M. Liu Y. (2022b). Mixed infections of plant viruses in nature and the impact on agriculture. Front. Microbiol. 13, 922607. doi: 10.3389/fmicb.2022.922607 35774460 PMC9238407

[B59] Xu L. Zhang W. Gao Y. Meng F. Nie X. Bai Y. (2022a). Potato virus y strain n-wi offers cross-protection in potato against strain NTN-NW by superior competition. Plant Dis. 106, 1566–1572. doi: 10.1094/PDIS-11-21-2539-SC 35072502

[B60] Ziebell H. Carr J. P. (2010). Cross-protection: acentury of mystery. Adv. Virus Res. 76, 211–264. doi: 10.1016/S0065-3527(10)76006-1 20965075

