# Supplementary material for: Virus-virus interactions alter the mechanical transmissibility and host range of begomoviruses
Source: Front Plant Sci. 2023 Jun 2;14:1092998. doi: 10.3389/fpls.2023.1092998 (PMC10275492; doi:10.3389/fpls.2023.1092998)
Supplement: Supplementary file 1 [file Table_1.docx]

**Table S1** List of primers used in this study

| **Primer** | **Sequence** | **Function** |
| --- | --- | --- |
| FJJ2010-67 | 5’-GTGGGATCCATTATTGCAC | For DNA A of ToLCNDV-OM detection |
| FJJ2010-92 | 5’-tacggggaataaagaagatgcg |  |
| FJJ2010-59 | 5’- GTTTATGACATGAATGAAATTGTG | For DNA B of ToLCNDV-OM detection |
| FJJ2010-60 | 5’- TAGCGATTGGACAATACAC |  |
| FJJ2011-70 | 5’- CTCTATGTAATTGGTGTCTGGA | For DNA A of ToLCNDV-CB detection |
| FJJ2017-134 | 5’-TGGCACGTCGGGACTTCTAT |  |
| FJJ2018-14 | 5'-CACCATGTCAATAGGAAATGAT | For DNA B of ToLCNDV-CB detection |
| FJJ2018-16 | 5'-TTACACGCCCTTTGATATTGGA |  |
| FJJ2009-1 | 5’-TAGTTCCCCGGTGCGTAAATCCAT | For DNA A of TYLCTHV detection |
| FJJ2009-2 | 5’-ACATCCCCAACCAGGTCAGCACAT |  |
| FJJ2009-3 | 5’-CCTCCATGGATTCGGGTCTTTG | For DNA B of TYLCTHV detection |
| FJJ2009-4 | 5’-CTGCGCGGCCTGATTTCCATAA |  |
| FJJ2016-84 | 5’-CTCTGCTTGAGCTGCAGTG | For DNA of ToLCTV detection |
| FJJ2005-85 | 5’-GGTCTCTAGGCCCACACAAT |  |
| FJJ2018-13 | 5’-CACCATGTCAACAGGAAATGAA | For MP gene of ToLCNDV-OM construction |
| FJJ2018-17 | 5’-CACGCCCTTTGATATTGGA |  |
| FJJ2020-119 | 5’-CACCATGGAGTCCAGAACTAACAAT | For MP gene of TYLCTHV construction (c-Myc tag was underlined) |
| FJJ2020-120 | 5’-CAGATCTTCTTCAGAAATAAGTTTTTGTTC TATTTTCTTTGCATTAGAAGAGAC |  |
| FJJ2021-217 | 5’-CACCATGGCTCTTTCTTCTCCTT | For NSP gene of ToLCNDV-OM construction (HA tag was underlined) |
| FJJ2021-218 | 5’-AGCGTAGTCTGGGACGTCGTATGGGTA TCCAATGTAATTAAG |  |
| FJJ2020-117 | 5’-CACC ATGGTTTCGTCGTTG | For NSP gene of ToLCNDV-CB construction (HA tag was underlined) |
| FJJ2020-118 | 5’-AGCGTAGTCTGGGACGTCGTATGGGTA TCCAATGTAATT |  |
| FJJ2020-115 | 5’-CACCATGTCGAAGCGTCCC | For CP gene of ToLCTV construction (HA tag was underlined) |
| FJJ2020-116 | 5’-AGCGTAGTCTGGGACGTCGTATGGGTA ATTTTGAACAGA |  |
